# Psychological Impact of COVID-19 and Its Influence on Parental Willingness to Vaccinate Children: A Cross-Sectional Study at a Tertiary Care Hospital in Kolkata

**DOI:** 10.7759/cureus.49585

**Published:** 2023-11-28

**Authors:** Jayeeta Burman, Nivedita Roy Choudhury, Amitabha Chattopadhyay, Sembagamuthu Sembiah, Anubrata Karmakar, Mukesh Shukla

**Affiliations:** 1 Community Medicine, Sarat Chandra Chattopadhyay Government Medical College & Hospital, Uluberia, IND; 2 Community Medicine, Nil Ratan Sircar Medical College and Hospital, Kolkata, IND; 3 Community Medicine, Midnapore Medical College and Hospital, Midnapore, IND; 4 Community Medicine and Family Medicine, All India Institute of Medical Sciences, Kalyani, Kalyani, IND; 5 Community Medicine, North Bengal Medical College and Hospital, Siliguri, IND; 6 Community and Family Medicine, All India Institute of Medical Sciences, Rae Bareli, Raebareli, IND

**Keywords:** covid_19 vaccination, parental distress, psychological impact of covid-19, covid-19 vaccine hesitancy india, covid-19 india

## Abstract

Background

COVID-19 has affected the physical and mental health of people globally, and vaccination is seen as a crucial tool in controlling the pandemic. However, the readiness to vaccinate children remains a concern, particularly in India.

Aim

The study aimed to investigate the association between the psychological impact of COVID-19 and willingness to vaccinate their children among attendees of the COVID-19 vaccination clinic at Nil Ratan Sircar Medical College, Kolkata.

Method

The study used an observational, cross-sectional design and collected data from 356 participants between August and September 2022. The COVID-19 Perceived Stress Scale-10 was used to assess participants' psychological impact, and willingness to vaccinate was determined using a survey.

Results

Approximately 64% (n=227) and 71% (n=253) of the participants exhibited a high level of perceived stress and willingness to vaccinate their children. The vaccine acceptance was significantly associated with perceived stress level and other factors such as family type, presence of chronic illness, and history of acquaintances suffering from COVID-19.

Conclusion

The study highlights the importance of addressing parental stress and anxiety to enhance vaccination rates among children. To achieve this, population-level awareness of vaccine safety measures and benefits should be raised to alleviate stress and increase vaccine uptake.

## Introduction

The COVID-19 pandemic has caused a significant global public health crisis, with millions of people affected worldwide. As the world grapples with the challenges posed by this deadly virus, the development and deployment of vaccines have emerged as critical tools in controlling its spread and ultimately achieving herd immunity. However, the success of vaccination campaigns heavily relies on the willingness of individuals to get vaccinated, and parental attitudes toward vaccines play a crucial role in this context [1,2]. Studies have shown that vaccine hesitancy is a significant barrier to achieving herd immunity and ending the COVID-19 pandemic [3].

The psychological impact of COVID-19 has also been widely documented, with the pandemic having a significant impact on the mental health of people worldwide [4]. Parental attitudes towards vaccines have been identified as an important determinant of vaccination behavior. Previous research has shown that parents' perceptions of the safety and efficacy of vaccines, as well as their trust in healthcare providers, influence their willingness to vaccinate their children [5]. However, psychological health seems to be a controversial determinant of vaccine hesitancy and remains to be investigated.

Therefore, this study aims to investigate the association between psychological impact and parental willingness to vaccinate their child against COVID-19 among attendees of the COVID-19 vaccination clinic at a tertiary care hospital in Kolkata. Gaining insights into the complex interplay between psychological well-being and vaccination behavior could enable the development of targeted interventions and communication strategies. By addressing specific psychological concerns and tailoring vaccine promotion efforts accordingly, health authorities can bolster vaccination rates and enhance public health outcomes in the fight against COVID-19.

## Materials and methods

This study is an observational cross-sectional study conducted among attendees of the COVID-19 vaccination clinic of a tertiary care hospital in Kolkata, India from August to September 2021. An international survey revealed that 65% of caregivers expressed their intention to vaccinate their child against COVID-19 [6]. With a prevalence(p) of 65%, an absolute error of 5%, and a confidence level of 95%, the sample size was calculated using the formula = z2 pq/d2 (z = standard normal deviate at 95% confidence interval [CI]; q = 100 - p), the minimum sample size came out to be 350. The participants were selected through purposive sampling. Ethical approval was obtained from the Institutional Ethics Committee of Nil Ratan Sircar Medical College & Hospital vide Letter No. NMC/6422 dated 21/10/2021, and informed written consent was taken from all participants. The study was conducted in accordance with the Declaration of Helsinki.

The study used a pre-designed, pre-tested, structured interview schedule containing open- and close-ended questions to collect participant data. The tool was translated into Bengali and back to English, and the face and content validity of the instrument was checked by the experts of the institutions.

The dependent variable was the parents’ willingness to vaccinate their children under the age of 18 with a COVID-19 vaccine using a question; the responses were “Yes”, “No” and “Not sure”; followed by an open-ended question “Why?” or “Why not?”, with a free text box.

The main independent variable was the psychological impact due to COVID-19, which was assessed using the COVID-PSS-10, used to assess the perception of stress associated with COVID-19 [7]. This validated tool includes questions about feelings and thoughts during the last month, and participants were asked to indicate how often they felt or thought in a certain way. COVID-PSS-10 is made up of 10 items, each of which offers five response options: never, almost never, occasionally, almost always, and always. COVID-PSS-10 presented a bi-dimensional construction: the first section named 'Stress' focused on the perceived lack of control over the situation (question numbers 1, 2, 3, 6, 9, and 10) ranging from almost always with 4 points to never with 0 points. The second section named 'Coping' defined a set of cognitive and behavioral strategies needed to manage stressful situations (question numbers 4, 5, 7, and 8) which were inversely scored.** **The total scores obtained ranged from a minimum of 0 to a maximum of 40. Scores at or above 25 were interpreted as the high perceived stress associated with COVID-19.

The other independent variables were socio-demographics such as age, gender, residence, educational status, monthly income, occupation, family type, addiction, and co-morbidity data. Additionally, knowledge on COVID-19 was collected using a set of questions prepared from the literature review [8].

Ethical approval from the institutional ethical committee was obtained and informed written consent was obtained from all participants in the study. The study was conducted in accordance with the declaration of Helsinki.

Data were entered in Microsoft Excel 2007 (Microsoft Corporation, Redmond, USA) and analyzed using the SPSS software version 16.0 (SPSS Inc., Chicago, USA). Logistic regression was used to elicit the relationship between perceived stress among parents and their willingness to vaccinate their children for COVID-19. We then used multivariable logistic regression analysis to estimate the adjusted odds ratio of agreeing to vaccinate children, using all the variables that showed significance (p < 0.05) in the univariate analysis.

## Results

Among 356 participants, most (211, 59%) of the participants belonged to the age group 18-45 years (Mean ± SD → 41.32 ± 17.1). Males were more in number than females. About half (187, 52.5%) of the participants were graduates. About half (182, 51.1%) of participants’ acquaintances were affected by COVID-19 infection (Table 1).

**Table 1:** Distribution of study participants according to demographic profile and general information (n=356)

Characteristics	n(%)
Age in years	
18-45	211(59.3)
46-60	93(26.1)
>60	52(14.6)
Gender	
Male	222(62.4)
Female	134(37.6)
Education	
Primary	43(12.1)
High school	72(20.2)
Intermediate	54(15.2)
Graduate	187(52.5)
All of the above	208(59.4)
Type of Family	
Nuclear	176(49.4)
Joint	180(50.6)
Occupation	
Homemaker / Unemployed	151(42.4)
Unskilled worker	44(12.4)
Skilled worker	13(3.6)
Clerical/ shop/ Farmers	25(7)
Professional	133(34.6)
Chronic illness	
Yes	120(33.7)
No	236(66.3)
Acquaintances affected by COVID-19	
Yes	182(51.1)
No	174(48.9)

Nearly two-thirds (227; 64%) of the participants had a high level of perceived stress level (scores >25) (Table 2).

**Table 2:** Distribution of study participants according to the psychological impact of COVID-19 (n=356)

Statements	Never [n(%)]	Hardly ever [n(%)]	Occasion [n(%)]	Almost always [n(%)]	Always [n(%)]
I have felt as if something serious was going to happen unexpectedly with the epidemic	17(4.8)	40(11.2)	57(16)	115(32.3)	127(35.7)
I have felt that I am unable to control the important things in my life because of the epidemic	63(17.7)	57(16)	127(35.7)	65(18.3)	44(12.4)
I have felt nervous or stressed about the epidemic	75(21.1)	64(18)	64(18)	49(13.8)	104(29.2)
I have been confident about my ability to handle my personal problems related to the epidemic	61(17.1)	47(13.2)	127(35.7)	109(30.6)	12(3.4)
I have felt optimistic that things are going well with the epidemic	36(10.1)	67(18.8)	156(43.8)	85(23.9)	12(3.4)
I have felt unable to cope with the things I have to do to monitor for a possible infection	64(18)	46(12.9)	80(22.5)	136(38.2)	30(8.4)
I have felt that I can control the difficulties that could appear in my life as a result of the infection	57(16)	45(12.6)	121(34)	88(24.8)	45(12.6)
I have felt that I have everything under control in relation to the epidemic	49(13.8)	113(31.8)	71(19.9)	68(19.1)	55(15.4)
I have been upset that things related to the epidemic are out of my control	53(14.9)	39(11)	11(31.2)	36(10.1)	117(32.9)
I have felt that the difficulties are increasing in these days of the epidemic and I feel unable to overcome them	75(21.1)	42(11.8)	82(23)	64(18)	93(26.1)

About three-fourths (253; 71%) proportion of participants were willing to vaccinate their children. The most common reason reported by caregivers willing to vaccinate was to protect their child (207, 82%) followed by the severity of the pandemic (190, 75%), and lastly, 7% (n=18) said that the vaccines work in preventing the disease. The most common reason reported by caregivers refusing vaccination was that the vaccine is new and there was no adequate background information about the effectiveness of the vaccine (84, 82%), followed by concerns over side effects and the safety of taking the vaccines (78,76%) (Table 3).

**Table 3:** Distribution of study participants according to their willingness to vaccinate their children (n = 356) *Multiple responses

	n(%)
Willing to vaccinate their children	253 (71)
Reasons:*	
Children and family members are at higher risk	207 (82)
Severity of COVID -19	190 (75)
Everyone accepting the vaccine	25 (10)
Vaccines are effective in preventing the infection	18 (7)
Not willing to vaccinate	103 (29)
Reasons:*	
New vaccine; effectiveness not known	84 (82)
Side effects and safety concerns	78 (76)
COVID-19 is not a serious problem	13 (13)
Already infected, hence vaccine is not required	4 (4)

The univariate logistic regression found a significant association between parents' perceived stress level due to COVID-19 and their willingness to vaccinate their children against COVID-19. Other factors such as family type, occupation, and suffering from chronic illness were also associated. In multivariable logistic regression, all the factors remained significant. With the Hosmer-Lemeshow test being non-significant and the Naglerke R2 value, the model is considered fit and is able to explain about 18% variability in the willingness to vaccinate their children (Table 4).

**Table 4:** Logistic regression to assess the relationship between stress level and willingness to vaccinate their children OR = Odds ratio; AOR = Adjusted Odds Ratio

Variables	Willingness to vaccinate their child	
	OR (95% CI); P value	AOR (95% CI); P value
Perceived stress (high)	2.6 (1.5 – 4.5); 0.003	1.7 (1.4-3.1) 0.004
Age (>45 years)	1.06 (0.6-1.6); 0.82	
Gender (Male)	1.2 (0.5-2.8); 0.51	
Education (Graduation and above)	1.3 (0.8-2); 0.25	
Family type (Joint)	3.5 (2.3-5.8); <0.001	2.9 (1.7-6.8); <0.001
Occupation (Working population)	3.5 (2.1-5.6); <0.001	2.7 (1.3-8.2); <0.001
History of chronic illness	7.1 (3.5-14.7); 0.001	6.7 (3.2-21.2); 0.001
Acquaintances affected by COVID-19	2.1 (0.9-3.1); 0.45	
Hosmer-Lemeshow test		0.8
Nagelkerke R^2^		0.18

## Discussion

A globally implemented, safe vaccination program is considered one of the key long-term solutions to effectively control and mitigate the coronavirus disease 2019 (COVID-19) pandemic. Vaccination programs can have significant clinical and socioeconomic benefits by reducing the spread of the virus, preventing severe cases and hospitalizations, and ultimately saving lives. Vaccinating children against COVID-19 will play a crucial role in containing the disease's spread, as achieving herd immunity might necessitate vaccinating around 80% of the population [9].

The current study aimed to investigate the association between the psychological impact of COVID-19 and the willingness to vaccinate children among attendees of a COVID-19 vaccination clinic. The study found that nearly two-thirds of the participants had a high level of perceived stress level, and this stress level was associated with their willingness to vaccinate their children.

The finding of high perceived stress levels among participants is consistent with previous studies that reported a significant increase in psychological distress and mental health problems during the COVID-19 pandemic [10,11]. For example, a study conducted in China found that the prevalence of anxiety and depression symptoms among the general population was as high as 35% and 20%, respectively [12]. Another study conducted in India reported that more than 40% of the respondents reported moderate to severe anxiety levels during the pandemic [13].

The study also found that family type, presence of co-morbidity, and history of acquaintances suffering from COVID-19 were associated with the perceived stress level of the participants. This finding is consistent with previous research that has reported a significant association between COVID-19-related stress and knowledge, perception, and attitudes towards the pandemic [14]. This finding underscores the need for tailored communication strategies and education programs that address the specific concerns and stressors of different subgroups of parents.

Moreover, the study found that 71% of participants were willing to vaccinate their children against COVID-19. This is consistent with previous studies that have reported a high willingness to vaccinate children against COVID-19 among parents [9, 15]. The present study demonstrates a significant association between parents' perceived stress level due to COVID-19 and their willingness to vaccinate their children against COVID-19. This finding is consistent with previous studies that have shown how stress and anxiety can impact health behaviors, including vaccination uptake [15,16]. These studies support the idea that parents who experience higher levels of stress related to COVID-19 may be more motivated to prioritize their children's health and well-being, including by vaccinating them against COVID-19 and other illnesses. However, it's important to consider the complex interplay between stress, health beliefs, and vaccine attitudes when interpreting these findings. Future research should further explore the underlying mechanisms linking stress and vaccine hesitancy, as well as identify effective strategies for reducing parental stress levels and increasing vaccine uptake. The public’s positive views and trust in a fast-tracked COVID-19 vaccine will be essential because some individuals are unsure about its safety and necessity due to concerns surrounding new vaccines.

The study had some limitations. It was cross-sectional in design, limited to one geographic setting, and did not consider the characteristics of the children. Additionally, the study relied on self-reported data, which may be subject to response bias and social desirability bias.

## Conclusions

This study examined the link between parents' perceived stress levels due to COVID-19 and their willingness to vaccinate their children. The findings revealed a significant association, highlighting the importance of addressing parental stress and anxiety to enhance vaccination rates among children. To achieve this, population-level awareness of vaccine safety measures and benefits should be raised to alleviate stress and increase vaccine uptake. Public health officials play a crucial role in this process by providing evidence on COVID-19 vaccines' safety and efficacy, emphasizing the risks and consequences of infection in children, and educating caregivers on the role of vaccination. Incorporating these factors into vaccination campaigns and communication strategies can effectively encourage parents to consider vaccinating their children against COVID-19, thus contributing to overall public health efforts to combat the pandemic.
